# Supplementary material for: Comparative multiomics analysis of cell physiological state after culture in a basket bioreactor
Source: Sci Rep. 2022 Nov 23;12:20161. doi: 10.1038/s41598-022-24687-4 (PMC9686226; doi:10.1038/s41598-022-24687-4)
Supplement: Supplementary file 1 — Supplementary Information 1. [file 41598_2022_24687_MOESM1_ESM.zip › raw data/Transcriptomic raw data/result_tree.html]

Directory Tree


# Directory Tree

../Result\_X101SC22032454-Z02-F001-B1-16
  
 ├── 0.SupFile：0号文件夹为参考基因序列与注释准备文件夹
  
 │   └── GOclassification：GO注释  
 ├── 1.QC：测序数据质量评估
  
 │   ├── 1.Error：测序错误率分布检查  
 │   ├── 2.GC：A/G/C/T含量分布检查  
 │   ├── 3.Filter：测序数据过滤（clean reads、低质量、含N等所占比例）  
 │   └── 4.Stat：测序数据质量情况汇总（raw、clean、Q20、Q30、GC等）  
 ├── 2.Mapping：参考基因组比对
  
 │   ├── 1.Region：样本比对区域分布图  
 │   └── 2.Stat：样本比对率统计表格  
 ├── 3.Assemble
  
 ├── 4.Quant：基因定量分析结果
  
 │   ├── 1.Count：样本各基因readcount、FPKM和组的各基因FPKM  
 │   ├── 2.Correlation：各样品相关性系数图、主成分分析PCA二维/三维聚类图  
 │   └── 3.Distribution：不同表达水平区间的基因数量统计表和相应的分布图、小提琴图以及盒型图  
 ├── 5.Differential：差异表达分析
  
 │   ├── 1.deglist：1、差异分析列表 2、差异显著表达基因列表（分为全部、上调、下调）3、差异基因火山图  
 │   ├── 2.venn：韦恩图  
 │   └── 3.cluster：聚类热图  
 ├── 6.Enrichment：差异基因富集分析
  
 │   ├── 1.GO：差异基因GO（功能分类）富集分析  
 │   ├── 2.KEGG：差异基因KEGG（代谢通路）富集分析  
 │   └── 3.PPI：蛋白互作网络分析（只提供差异基因蛋白互作网络数据文件(cytoscape)）  
 ├── 7.AS：可变剪切分析
  
 │   ├── 1.daslist：差异可变剪接事件  
 │   └── 2.dasplot：可变剪接可视化  
 └── 8.SNP：SNP和Indel分析
  
     ├── 1.snpsite：变异位点检测  
     ├── 2.snpeff：变异位点注释  
     └── 3.stat：变异位点统计

30 directories

---

tree v1.5.3 © 1996 - 2009 by Steve Baker and Thomas Moore   
HTML output hacked and copyleft © 1998 by Francesc Rocher   
Charsets / OS/2 support © 2001 by Kyosuke Tokoro
